# Genetic variability of Akhal-Teke horses bred in Italy

**DOI:** 10.7717/peerj.4889

**Published:** 2018-09-06

**Authors:** Maria C. Cozzi, Maria G. Strillacci, Paolo Valiati, Elisa Rogliano, Alessandro Bagnato, Maria Longeri

**Affiliations:** Department of Veterinary Medicine, Università degli Studi di Milano, Milan, Italy

**Keywords:** Horse, Akhal-Teke, mtDNA D-loop, Microsatellite markers, Genetic variability, Biodiversity, Sequencing, Median joining network, Neighbor-joining tree, PCA

## Abstract

**Background:**

The Akhal-Teke horse (AKH) is native of the modern Turkmenistan area. It was introduced in Italy from 1991 to 2000 mainly as an endurance horse. This paper characterizes the genetic variability of the whole Italian AKH horse population and evaluates their inbreeding level by analyzing microsatellite markers and mitochondrial D-Loop sequences.

**Methods:**

Seventeen microsatellite marker loci were genotyped on 95 DNA samples from almost all the AKH horses bred in Italy in the last 20 years. Standard genetic variability measures (H_o_, H_e_, F_IS_) were compared against the same variables published on other eight AKH populations. In addition, 397 bp of mtDNA D-loop region were sequenced on a sub-group of 22 unrelated AKH out of the 95 sampled ones, and on 11 unrelated Arab horses. The haplotypes identified in the Italian population were aligned to sequences of AKH (56), Arab (five), Caspian Pony (13), Przewalskii (two) and Barb (15) horses available in GenBank. The Median Joining Network (MJN), Principal Component Analysis (PCA) and Neighbor-joining (NJ) tree were calculated on the total 126 sequences.

**Results:**

Nucleic markers showed a high degree of polymorphism (H_o_ = 0.642; H_e_ = 0.649) and a low inbreeding level (F_IS_ = 0.016) in Italian horses, compared to other AKH populations (ranged from −0.103 AKH from Estonia to 0.114 AKH from Czech Republic). High variability was also recorded in the D-Loop region. 11 haplotypes were identified with haplotype diversity (hd), nucleotide diversity (*π*) and average number of nucleotide differences (k) of 0.938, 0.021 and 6.448, respectively. When all the 126 D-Loop sequences were compared, 51 haplotypes were found, and four were here found only in the Italian AKH horses. The 51 haplotypes were conformed to eight recognized mtDNA haplogroups (A, C, F, G, L, M, P and Q) and confirmed by MJN analysis, Italian horses being assigned to five haplogroups (A, C, G, L and M). Using a PCA approach to the same data, the total haplotypes were grouped into two clusters including A+C+M+P and G+F haplogroups, while L and Q haplogroups remained ungrouped. Finally, the NJ algorithm effectively discretizes only the L haplogroup. All the above data univocally indicate good genetic variability and accurate management of the Akhal-Teke population in Italy.

## Introduction

In the recent past, several molecular markers and techniques have been used to investigate the genetic origin and variability within and across local endangered populations to disclose biodiversity and to support conservation plans (Cothran, van Dyk & van der Merwe, 2001; Bömcke, Gengler & Cothran, 2011; Kusza et al., 2013).

Mitochondrial DNA (mtDNA) has also been widely used to estimate genetic diversity, phylogenetic relationships and maternal origins among closely related modern populations. Particularly for such purposes, the mtDNA D-loop hypervariable region 1 has been sequenced in equids (Beja-Pereira et al., 2004; McGahern et al., 2006; Royo et al., 2005; Prystupa et al., 2012; Zhang et al., 2012; Hristov et al., 2016; Sziszkosz et al., 2016; Cieslak et al., 2010; Cieslak et al., 2017; Cozzi et al., 2017). The distribution of haplogroups of the hypervariable region 1 and the complete sequence of the mtDNA D-loop from samples of horse remains from archaeological sites have been recently analyzed by several study teams to date horse domestication events (Jansen et al., 2002; Lippold et al., 2011; Achilli et al., 2012; Librado et al., 2017). These authors find a large number of maternal lineages in the domestic horse gene pool (Vilà et al., 2001; Cieslak et al., 2010). The geographic structuring of mtDNA is low today, but there is also evidence that some maternal lineages ware regionally confined (Cieslak et al., 2010). Recently, Elsner and collaborators (2017) tracked the dynamics of horse population migrations due to climate change in the Pleistocene-Holocene transition from nine archaeological sites adjacent to the Swiss Jura Mountains, providing a novel approach to the evolutionary study of the impact of human encroachment and habitat change on the development of horse populations.

Akhal-Teke (AKH) is a very ancient horse breed that is native of central Asia, precisely the southern region of modern Turkmenistan, and has adapted well for endurance capacity to cover long distances in harsh climate conditions of Central Asian steppes. The breed during the last 100 years has been selected strongly for speed (Leisson et al., 2011). As reported by several authors using protein markers, microsatellites, Single Nucleotide Polymorphisms (SNP) and mitochondrial DNA, the AKH belongs to the clade of Middle Eastern Breeds that includes Arabian, Caspian pony and Turkoman horses (Cothran, van Dyk & van der Merwe, 2001; Luis et al., 2007; Bömcke, Gengler & Cothran, 2011; Priskin et al., 2010; Achilli et al., 2012; Petersen et al., 2013).

The AKH breed is renowned for the distinguishing metallic shine of its hair. Moreover, natural athleticism makes a well-rounded sports horse of this breed, which is good at dressage, show jumping, eventing, racing, and endurance riding competitions. This ancient breed represents a unique genetic resource that differs from all the other breeds worldwide in terms of type, conformation and use. The pedigree recording started in 1885, and the Studbook was closed in 1932. The AKH was introduced in Italy from 1991 to 2000 mainly as an endurance horse.

This paper conducts a genetic variability analysis based on genomic DNA loci (microsatellite markers) and mtDNA D-loop of AKH horses reared in Italy. Furthermore, a meta-analysis was performed using horse mtDNA sequences available in GenBank, besides a comparison with literature data on the genetic variability of AKH horses bred in other countries.

## Material and Methods

### DNA extraction, genotyping and sequencing

The Italian AKH Association’s reference laboratory for genetic testing for parentage control at the Department of Veterinary Medicine (University of Milan) maintains the bio-bank of the biological samples of all AKH horses bred in Italy since they were first imported (107).

All applicable international, national and/or institutional guidelines for the care and use of animals were followed. The bio-banked samples were approved by the Ethics Committee of Università degli Studi di Milano Prot. OPBA_56_2016.

Ninety-five of the 107 blood samples (males and females) were genotyped using 17 microsatellite markers of StockMarks for the Horses^®^ Genotyping Kit (utilized for parentage testing), including 12 from the official ISAG panel (http://www.isag.us/Docs/EquineGenParentage2016.pdf). All PCR products were run on ABI PRISM^®^ 310 Genetic Analyzer, and fragments were analyzed with the Genescan^®^ and Genotyper^®^ software (http://tools.thermofisher.com/content/sfs/brochures/cms_040300.pdf).

According to the available pedigrees (first generation/parents), the horses with no common mare and stallion (*n* = 22) were sequenced, and so were 11 additional Arabian horses reared in Italy and bio-banked at the University of Milan. The 379 bp fragment of the mtDNA D-loop region (nucleotide position: 15,382–15,778) was amplified and sequenced by classic Sanger methods, using previously published primer pairs (Cozzi et al., 2004). The PCR products were purified utilizing SEPHADEX^®^ G-50 (SIGMA) and sequenced for both strands by using the Big Dye^®^ Terminator v.3.1 Cycle Sequencing Kit (Applied Biosystems, Foster City, CA, USA) according to the manufacturer’s guidelines. The PCR products were processed on an ABI PRISM^®^310 Genetic Analyzer, and the nucleotide sequence was determined using the ABI PRISM^®^ DNA Sequencing Analysis Software.

### Statistical analysis on microsatellite data

Allelic frequencies, genetic equilibrium according to Hardy–Weinberg (HW) (*P*-val), linkage disequilibrium (LD) and inbreeding coefficient (*F*_IS_) were estimated using the GENEPOP software (Raymond & Rousset, 1995). LD between a pair of loci was also tested for genotypic data using the likelihood-ratio test of Arlequin v.3.5.2.2 software (Excoffier & Lischer, 2010). The number of alleles (NA), the effective number of alleles (NE) and the observed (H_o_) and expected (H_e_) heterozygosity per locus and on the entire population were calculated with POPGENE v.1.32 (Yeh, Yang & Boyle, 1997). Allelic richness (AR) per locus and in the population was also used to estimate genetic population diversity. AR was standardized for variations in sample size, and was calculated using FSTAT 2.9.3 (Goudet, 2002), based on 79 diploid individuals with no missing genotypes.

### Statistical analysis on sequence data

Sequences were checked for the presence of ambiguous bases by using the software Chromas v.2.5.1 (http://www.technelysium.com.au/).

In order to define the haplotypes (H-) all obtained sequences were aligned with the complete horse mtDNA sequences X79547 (Xu & Arnason, 1994) and JN398377 (Achilli et al., 2012), widely used as reference sequences, using the ClustalW algorithm implemented in BioEdit (Hall, 1999).

The number of polymorphic sites, the parsimony informative and the singleton site, the number of haplotypes, the haplotype diversity (hd), the nucleotide diversity (*π*) and the average number of nucleotide differences (k) were estimated using the DnaSP5 v.5.10.01 software (Librado & Rozas, 2009). Haplotype pairwise genetic differentiation was calculated using Arlequin v.3.5.2.2 (Excoffier & Lischer, 2010).

In order to define the genetic relationships among our mtDNA sequences of AKH and those available in Genbank for other AKH lines and for other populations originated from the same Middle Eastern clade, a total of 104 sequences (two reference mtDNA, two Przewalski, five Arabian horse, 15 Barb horse and 13 Caspian pony, 56 Akhal-Teke and the 11 Arabian horses bred in Italy) were considered. All the sequences were trimmed at 254 bp to maximize the number of sequences included. Haplogroup definition was performed according to Achilli et al. (2012). The mtDNA sequences from GenBank database used for haplogroup definition and relationship investigation were reported in Table S1.

Three different approaches were used to investigate the haplotype and haplogroup relationship:

i) the Median-Joining Networks (MJN) analysis performed by Network v.5.0 software (http://www.fluxus-engineering.com); the default setting of Epsilon (0) was applied and the mutational hot spots present in the 254bp fragment were down-weighted as suggested by Jansen et al. (2002) and Cieslak et al. (2010);
ii) the Neighbor-joining (NJ) tree (Saitou & Nei, 1987) based on the Kimura-2-parameter model distances (Kimura, 1980) was calculated among the haplotypes with MEGA v.7.0.14 software (Kumar, Stecher & Tamura, 2016); the bootstrap analysis, running 1,000 bootstrap replicates, was applied to check the robustness of the resulting dendrogram and a cut-off bootstrap at a significant value of 50 was applied;
iii) the Principal Component Analysis (PCA) with a minimum spanning tree based on haplotype sequences was calculated using the PAST v.2.7 software (Hammer, Harper & Ryan, 2001).

The pairwise population genetic differentiation (*F*_ST_) was calculated by Arlequin v.3.5.2.2 (Excoffier & Lischer, 2010) and used to evaluate genetic differentiation among Italian AKH, Italian Arabian, and the 93 horse GenBank sequences.

All the horse mtDNA sequences obtained in this study were deposited (GenBank ID: MF580226–MF580244).

## Results

### Microsatellite markers

The results of microsatellite analyses are reported in Table 1. A total of 112 allelic variants were found on 17 microsatellites, ranging from four (HTG6 and HTG7) to 10 alleles (ASB2). The microsatellite LEX03 (nine alleles), mapping on X chromosome, was excluded from the statistical analyses.

**Table 1:** Measures of genetic variation in AKH bred in Italy by 16 microsatellites.

Locus	NA	NE	AR	*P*-val ± SE	H_**o**_	H_**e**_	F_**IS**_
AHT4	6	2.6	5.9	0.019 ± 0.01	0.653	0.617	−0.058
HMS7	6	2.6	5.9	0.676 ± 0.01	0.589	0.623	0.055
HTG4	6	1.7	5.8	0.026 ± 0.01	0.379	0.410	0.075
VHL20	9	3.8	8.8	0.442 ± 0.02	0.758	0.740	−0.024
AHT5	5	3.8	5.0	0.093 ± 0.01	0.755	0.740	−0.021
ASB2	10	5.3	9.8	0.511 ± 0.02	0.810	0.816	0.007
HMS6	6	2.8	5.8	0.139 ± 0.01	0.558	0.643	0.133
HTG6	4	2.2	3.9	0.071 ± 0.01	0.453	0.548	0.174
HMS2	8	3.6	7.8	0.429 ± 0.02	0.716	0.728	0.017
HMS3	7	2.9	6.9	0.154 ± 0.01	0.653	0.665	0.019
HTG10	7	3.5	6.8	0.346 ± 0.01	0.800	0.720	−0.113
HTG7	4	2.0	3.8	0.685 ± 0.01	0.505	0.493	−0.026
ASB17	9	5.2	8.9	0.280 ± 0.02	0.625	0.813	−0.061
ASB23	6	3.3	6.0	0.167 ± 0.01	0.600	0.702	0.147
CA425	6	2.1	6.0	0.015 ± 0.01	0.532	0.522	−0.018
HMS1	4	2.5	4.0	0.226 ± 0.01	0.642	0.609	−0.055
Mean ± SD	6.4 ± 1.8	3.1 ± 1.1	6.4	0.050 ± 0.01	0.642 ± 0.1	0.649 ± 0.1	0.016

**Notes.**

NA number of alleles NE effective number of alleles AR allelic richness HW Hardy-Weinberg *P*-val *P* values FIS observed (Ho) and expected (He) heterozygosity values and inbreeding coefficient values SE standard error SD standard deviation

The mean number of alleles (NA) calculated on 16 microsatellites was 6.4, whereas the effective mean number of alleles (NE) was 3.1. The analyses of the Hardy–Weinberg genetic equilibrium (*P*-val), calculated using HW formulae, showed three microsatellites (AHT4, HTG4 and CA425) that were not in genetic equilibrium (*P* < 0.05). This drift is probably caused by strongly unbalanced allelic frequencies in the small population originally imported to Italy from 1991 to 2000 (24 horses: 17 females and seven males).

According to the position (cM) of the microsatellites as reported by Swinburne et al. (2006), LD analyses were performed only for the markers located on the same chromosome: HMS6 and HTG7 (chr 4), HMS3 and HTG4 (chr 9) and ASB2, HTG6 and HMS1 (chr 15) (Table S2). LD between a pair of loci was tested in all samples together and by groups of individuals: those imported in Italy from 1991 to 2000 (Founders) and the ones born in Italy after 2000 (New population). Considering all samples together, all pairs of loci were in LD, except for HTG7 vs HMS6 (Table S2). The group of Founders was in equilibrium for all pair of loci; instead, for the New population the LD was observed for ASB2 vs HMS1 (GENEPOP and Arlequin) and ASB2 vs HTG6 (Arlequin) pairs of markers (Table S2). Hence, we calculated the NE based on the observed allelic frequencies that ranged from 1.7 (HTG4) to 5.3 (ASB2), thus revealing that some loci had a very unbalanced representation of allele variants. These included AHT4 and HTG4, two of the three loci that were not balanced, which present an NE value of 2.6 and 1.7, respectively.

Allelic richness (AR) is a parameter that is used to estimate diversity in the population. It ranges from 4 (HMS1) to 9.8 (ASB2), with a mean value of 6.4, comparable to the AR of 6.09 reported by Warmuth et al. (2011).

H_o_ and the H_e_ values ranged from 0.379 (HTG4) to 0.810 (ASB2), with a mean value of 0.642 and, from 0.410 (HTG4) to 0.816 (ASB2), with a mean value of 0.649, respectively The *F*_IS_ values per locus that ranged from −0.113 (HTG10) (outbreeding) to 0.174 (HTG6) (inbreeding); the overall *F*_IS_ value for the population was 0.016.

We estimated the diversity parameters on the AKH horses imported in Italy from 1991 to 2000. The values obtained were compared with those estimated in the AKH population born in Italy (after 2000). The two groups of horses are in HW equilibrium and have similar genetic values, except for the *F*_IS_ value that denotes, in the first nucleus, an apparent excess of heterozygosis Table S3.

### Mitochondrial DNA sequences

Table 2 shows the eleven haplotypes found in AKH reared in Italy (H1 to H11). The 22 polymorphic sites identified were 19 parsimony informative and three singleton sites (15495, 15538 and 15709). All the polymorphic sites reported in Table 2 were transition sites. The maximum composite likelihood estimate of the nucleotide substitution pattern, identified for the 22 sequences, were 32.5% (A), 26.6% (T), 29.1% (C), and 11.8% (G), depending on the order of nucleotide composition in the vertebrate mitochondrial genome (A>C>T>G). The A+T content at the mtDNA D-loop region was 59.1% and exceeded G+C content (40.9%). Zhang et al. (2012) for Chinese Native horse breeds and Sziszkosz et al. (2016) for Hungarian Gidran horses report similar rich A+T content values in horse mtDNA, precisely 55.8% and 64.1% respectively.

**Table 2:** Polymorphic sites in the horse Italian AKH mtDNA D-loop sequence nucleotide ranging from 15385 to 15862 positions, according to the reference sequences X79547 and JN398377.

Haplotypes	Position of nucleotide substitutions																						*N*	Freq.%
	15494	15495	15496	15521	15534	15538	15542	15585	15597	15602	15603	15604	15617	15635	15649	15650	15659	15666	15703	15709	15720	15737		
X79547	T	T	A	G	C	A	C	G	A	C	T	G	T	C	A	A	T	G	T	C	G	T	Reference	
JN398377	.	.	.	.	.	.	.	.	.	.	.	.	.	.	.	.	.	.	.	.	.	.	sequences	
H1	.	C	.	.	.	.	.	.	.	.	.	.	.	.	.	.	.	.	.	.	.	.	1	4.5
H2	.	C	.	.	.	.	.	.	.	T	.	.	C	.	.	.	C	.	.	.	A	.	3	13.6
H3	.	C	.	A	.	.	.	.	.	T	.	.	.	.	.	.	.	.	.	.	A	C	3	13.6
H4	.	C	.	.	.	.	T	A	G	T	.	.	.	T	.	G	.	A	C	.	A	.	4	18.2
H5	.	C	.	.	.	.	T	A	G	T	.	.	.	.	.	G	.	A	.	.	A	.	2	9.1
H6	.	C	.	.	.	.	.	.	.	T	.	.	.	.	.	G	.	.	.	.	A	.	1	4.5
H7	.	C	.	.	.	.	.	A	.	T	.	.	.	.	.	.	.	.	.	.	A	.	2	9.1
H8	.	C	.	.	.	G	.	A	.	T	.	.	.	.	.	.	.	.	.	T	A	.	1	4.5
H9	C	C	G	.	T	.	.	.	.	.	C	.	.	.	G	.	.	.	.	.	A	.	1	4.5
H10	C	C	G	.	T	.	.	A	.	T	C	A	.	.	G	.	.	.	.	.	A	.	3	13.6
H11	C	C	G	A	T	.	.	A	.	T	C	A	.	.	G	.	.	.	.	.	A	.	1	4.5

**Notes.**

N number of samples per haplotype Freq. % haplotypes frequency in percentage X79547 and JN398377 ID Reference sequences

Nucleotide differences compared to reference sequences ranged from 1 (H1) to 11 (H11). The most frequent haplotype found in our samples was H4 (18.2%), while the less frequent haplotypes were H1, H6, H8, H10 and H11 recorded in only one individual each (4.5%).

The values of variability parameter haplotype diversity (hd), nucleotide diversity (*π*) and average number of nucleotide differences (k) were 0.938, 0.021 and 6.448, respectively.

**Figure 1:**
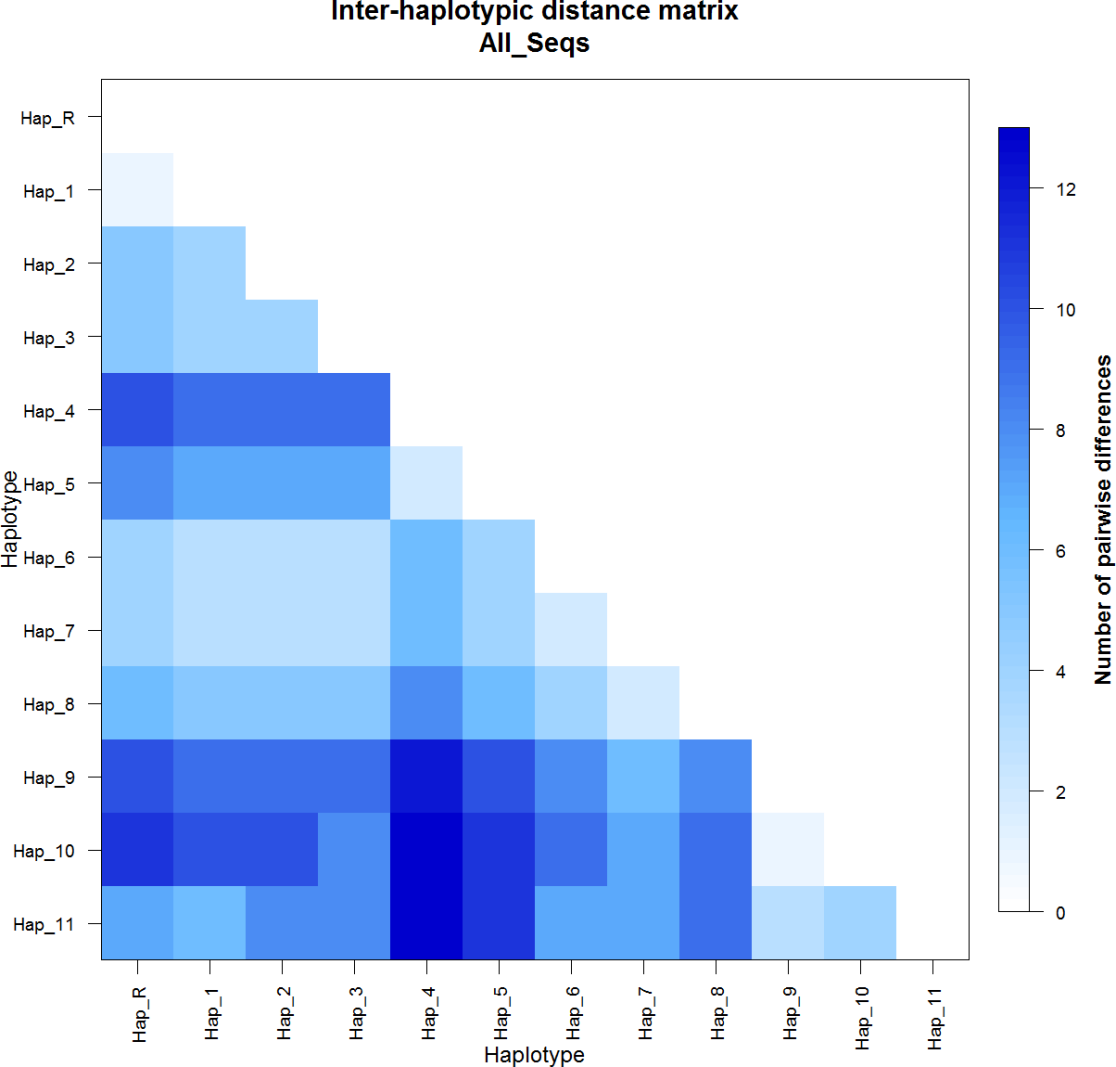
Inter-haplotypic pairwise distance matrix based on haplotypes pairwise genetic differentiation, with the legend of the color code. Hap_R = reference sequences X79547 and JN398377.

The pairwise distance matrix (Fig. 1) represents the number of nucleotide substitutions in each haplotype, where Hap_R represent the haplotype of the reference sequences. Inter-haplotypic differences ranged from 1 (Hap_R vs Hap_1 and Hap_10 vs Hap_11) to 13 (Hap_4 vs Hap_10 and Hap_4 vs Hap_11).

A total of 51 haplotypes with 36 variable sites (seven singleton and 29 parsimony informative) were identified by the alignment of the 126 mtDNA D-loop sequences, showing an hd value of 0.971. The detailed sequence variations of haplotypes and the identified haplogroups were reported in Table S4.

Four haplotypes (H16, H18, H21 and H22) were found exclusively in Italian AKH and were not reported in GenBank for the same breed. Additionally, our AKH sequences were grouped in five haplogroups A, C, G, L and M, as defined by Achilli et al. (2012).

**Figure 2:**
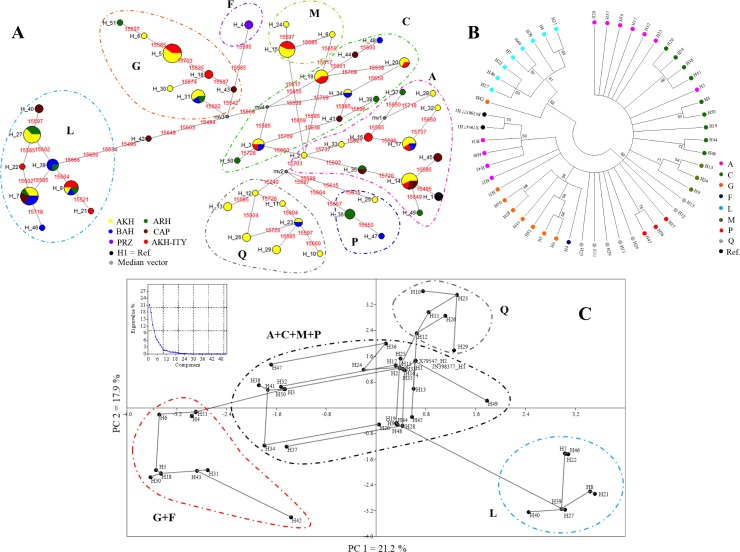
Clustering of haplogroups by MJN (A), NJ tree (B), PCA (C). (A) The haplotypes MJN based on the 124 horse mtDNA sequences and the two reference sequences (X79547 and JN398377). The numbers in red represent the position of nucleotide substitution; the black circle are the median vectors; circle are proportional to the numerosity of the haplotypes. AKH, Akhal-Teke; ARH, Arabian horse; BRH, Barb horse; CAP, Caspian Pony; PRZ, Przewalskii; H1, references. (B) The haplotypes NJ tree based on Kimura-2-parameter model distances. The numbers represent the robustness of the dendrogram after the cutoff bootstrap procedure was applied, with the legend of the haplogroup definition. (C) PCA analyses based on 51 haplotypes showing a minimum spanning tree. The graphic reports the percentage of variance explained by PCA components.

The MJN (Fig. 2A) showed the relationships among the 51 haplotypes occurring in the 126 mtDNA sequences, and discretized them in the eight haplogroups. Relationships among sequences were also defined in the NJ tree (Fig. 2B) and in the PCA analyses (Fig. 2C) that, nevertheless, are unable to dissect the variation among haplogroups as well as the MJN. Finally, the matrix of pairwise population genetic differentiation (*F*_ST_) showed a remarkable similarity between our AKH and other AKH populations, as expected (Table S4).

The NJ tree based on Kimura-2-parameter model distances and performed by applying a cut-off value of 50 for bootstrap value significance clearly separated haplogroups L from the others (62% of bootstrap value) (Fig. 2B). Haplogroups A and Q are divided into a different branch of the dendrogram (Fig. 2B). Particularly, haplotypes H2, which belong to haplogroup A, group together with haplotypes belonging to haplogroups C in another branch of the tree. The H2 located in the same operational taxonomic unit (OTU) of H3 and H50 differs from these by one mutation 15,650 (H2 *vs* H3) and by two mutations 15,650 and 15,728 (H2 *vs* H50), respectively. The haplotype H42, formally belonging to haplogroups G in the PCA but separated with haplogroup L in NJ tree, will be considered as intermediate among haplogroups G and L as well defined in MNJ (Figs. 2A, 2B and 2C).

The PCA analysis showing a minimum spanning tree based on sequence haplotypes, is displayed in Fig. 2C. The graphic placed in Fig. 2C reports the percentage of variance explained by PCA components. The first component (PC1) that explains most of the variability (21.2%) clearly separated haplogroups G+F and L, whereas the second component (PC2) (17.9%) defines haplogroup Q, confirming the MJN and NJ tree evidence. Haplogroups A, C, M and P appear mixed and are not mutually well separated both on PC1 and on PC2. The PCA analysis confirms the central role of H2.

## Discussion

In Italy, AKH horses are reared by few breeders and are mainly trained for shows and endurance races. The small number of animals and the use of the same stallions for reproduction entail a potential risk of decreasing variability. Hence, we analyzed genetic variability by using both microsatellite DNA nuclear markers and mitochondrial DNA variations.

As mentioned above, AKH belong to the clade of Middle Eastern Breeds that involved Arabian horses, Barb horses, Turkoman horses and Caspian pony. The native area of the AKH is considered one of the two hotspots for horse diversity. In fact, the Caspian Area of Western Asia served as refugium for wild horses in the early and mid-Holocene when, due to a lack of suitable habitat as a result of forest expansion, the wild horses migrated to more comfortable areas for this steppe-adapted species (Warmuth et al., 2011). Later, around 3500 BC, the Caspian Area and the Kazakh steppe were horse domestication points from where horses spread across Eurasia (Outram et al., 2009; Cieslak et al., 2010; Warmuth et al., 2012).

The mean number of alleles (MNA) (6.5) obtained in Italian AKH was comparable to those reported by Szontagh et al. (2005) for the AKH population bred in Turkmenistan and in Turkoman horses, using the same panel of 12 microsatellites (Table 3).

**Table 3:** Comparison with results reported by Szontagh et al. (2005)^a^ and by Jiskrová, Vrtková & Prausová (2016)^b^ using the same panel of 12 and 13 microsatellites.

Breed	NSTS	NS	NA	MNA	NE	H_**o**_	H_**e**_	F_**IS**_
*Akhal-Teke Italy*	*12*	*95*	*78*	*6.5*	*3.07*	*0.636*	*0.645*	*0.009*
Akhal-Teke Turkmenian^a^	12	48	73	6.1	4.03	0.664	0.703	0.053
Akhal-Teke USA^a^	12	58	91	7.6	4.03	0.655	0.727	0.100
Akhal-Teke Iran^a^	12	19	70	5.8	4.20	0.728	0.725	−0.005
*Akhal-Teke Italy*	13	*95*	*82*	*6.3*	*3.03*	*0.636*	*0.642*	*0.004*
Akhal-Teke Czech Republic^b^	13	121	114	8.8	4.12	0.648	0.731	0.114
Akhal-Teke Russia^b^	13	152	74	5.7	3.43	0.669	0.668	−0.005
Akhal-Teke Estonia^b^	13	28	62	4.8	3.16	0.720	0.665	−0.103
Akhal-Teke Switzerland^b^	13	24	63	4.8	3.04	0.664	0.656	−0.029

**Notes.**

NSTS number of microsatellites NS number of samples NA absolute number of alleles MNA Mean number of alleles NE effective number of alleles H_o_ observed heterozygosity H_e_ expected heterozygosity F_IS_ inbreeding coefficient

The NE value indicates the actual impact of alleles on population variability, precisely, a small value reports an unbalanced frequency of the alleles at a certain locus. The NE is associated to the expected heterozygosity. Indeed, heterozygosity is highest when allelic frequencies are balanced, and every allele contributes to the absolute number of alleles detected per locus.

In the Italian AKH horses we identified a lower effective number of alleles (NE) compared to the other AKH populations. Indeed, in the Italian population the founder effect is still having a strong impact on the population’s genetic composition due to the low number of stallions and dams imported from abroad to constitute the first Italian nucleus in 1991–2000 **(Founders)**. Particularly, the low number of stallions used in the last twenty years by breeders appears to have strongly influenced allele frequencies, as can be derived by the deviation from HW equilibrium for some microsatellite markers as shown in Table 1.

Several authors reported the influence of inbreeding, founder effect and population subdivision on LD. Nevertheless as pointed out by Farnir et al. (2000), McRae et al. (2002) and Miller et al. (2015) after one generation, it can be observed that the major part of the alleles in most of the offspring could be in the same phase as in the parents. The LD here found suggests a possible bottleneck effect occurred when the Founder group has been imported in Italy (Table S2). Despite that, the Italian Akhal-Teke population maintained a large genetic variability and low degree of inbreeding over the years.

The MNA value for Italian AKH, comparing with values obtained by Jiskrová, Vrtková & Prausová (2016) using a panel of 13 microsatellites, is lower respect to the Czech Republic AKH (Table 3). Additionally, the effective number of alleles is similar to those found by Jiskrová, Vrtková & Prausová (2016) in the small AKH population bred in Estonia and in Switzerland (Table 3).

The overall *F*_IS_ values obtained using the 17 microsatellites panel (0.016) and both 12 and 13 microsatellites panels (0.009 and 0.004), revealed a low level of inbreeding, i.e., the Turkmenian population, indicating the fact that Italian breeders are prone to avoid mating between related individuals as much as possible. This trend can be highlighted by comparing the *F*_IS_ values in Italian horse to other populations (Table 3). US and Czech AKH horses appear to have a higher level of inbreeding, compared to Italian AKH, which reflect good breeding practice of Italian breeders.

Finally, H_o_ and H_e_ in the Italian population are similar to those observed in the other AKH populations (Table 3), showing that, overall, selection pressure for endurance and athleticism is not intense and genetic variability appear to be conserved in the worldwide AKH population.

The results of Italian AKH mtDNA analyses reveal the presence of 11 haplotypes with a high haplotype diversity (hd = 0.938) and a high nucleotide diversity (*π* = 0.021), indicating a high level of variability in the Italian population. Many authors report similar results for several horse breeds, specifically, in modern AKH (hd = 0.94; *π* = 0.023) (Priskin et al., 2010), Hungarian Gidran horse (hd = 0.913; *π* = 0.021 (Sziszkosz et al., 2016), Anatolian horse (hd = 0.98; *π* = 0.026) (Koban et al., 2012), Caspian horse (hd = 0.96; *π* = 0.019) (Prystupa et al., 2012) and in Chinese horse breeds (hd range 0.97–0.98; *π* range 0.019–0.024) (Zhang et al., 2012).

The high genetic variability of mtDNA is proof of the broad genetic base of founder mares in the AKH population, though only the sire lines were historically documented (http://www.akhal-teke.org/at-lines.html), whereas no information was recorded for the female lines.

As expected, we found a great similarity between our AKH sequences and all the AKH sequences from GenBank, in contrast with Priskin et al. (2010), who identified significant differences in *F*_ST_ values (*F*_ST_ = 0.075, *P* = 0.027) among modern AKH, and the 18 Kazakh AKH deposited by McGahern et al. (2006) in GenBank (Table S5). It is interesting to note the low *F*_ST_ values found with one of the two Caspian Pony populations (AKH_ITY *vs.* CAP_a *F*_ST_ = 0) and with the two Arab horse populations (AKH_ITY *vs.* ARH_a *F*_ST_ = 0; AKH_ITY *vs.* ARH_ITY *F*_ST_ = 0.027) (Table S5). These small *F*_ST_ values confirmed that AKH belong to the same phylogenetic group of the Caspian Pony and Arab horse as reported by several authors (Cothran, Juras & Macijauskiene, 2005; McGahern et al., 2006; Cieslak et al., 2010; Achilli et al., 2012).

Haplotype H2, which belongs to haplogroup A, both on MJN and on PCA, appears to be the central point, and it is the link among all the haplogroups (Fig. 2A and 2C). The central role of H2 is due to the two parsimony sites 15,602 and 15,720 and the singleton site 15,495 that are diagnostic mutational motives for more than one haplogroup (McGahern et al., 2006; Achilli et al., 2012).

Haplogroups A, C, M, P and Q are clearly separated on the MJN, but the relationships among these haplogroups are not well defined in the PCA and in the NJ tree. In fact, the PCA is able to separate only the haplogroup Q form others, whereas in the NJ tree they are mixed with the P haplogroup (Fig. 2B and 2C). The 22 mtDNA sequences of Italian AKH horses were grouped in the five haplogroups A, C, G, L and M.

Consistently with literature, (McGahern et al., 2006; Cieslak et al., 2010; Achilli et al., 2012), haplogroup A **is** the most frequent (23.5%) in modern Middle Eastern horse populations as well as in the Western central Eurasia, one of the primary areas of horse domestication event, that comprises the AKH origin area, (Warmuth et al. (2012). In agreement with Achilli et al. (2012)), haplogroup F was represented only by the two Przewalskii samples sequenced (1.9% of analyzed sequences). The separation of haplogroup F from others appears clear in the MJN, while it is grouped with G in NJ and PCA analyses.

The four median vectors identified by haplogroups C-G and G-L should be interpreted as incomplete sequences that limited the connection under the maximum parsimony hypothesis and it could be explained by the presence of ancestors not included in the analyses and with extinct maternal sequences. In fact, without the median vector the shortest connection among analyzed sequences would be lost (http://www.fluxus-engineering.com/Network5000_user_guide.pdf). Hence, the haplotype H42, which formally belonged to haplogroups G, has to be considered as intermediate among haplogroups G and L (Fig. 2A). The three different methods used to evaluate the genetic relationships among haplogroups clearly indicate that haplogroup L is separated from the other by 4 parsimony informative mutations. In the overall mtDNA sequences considered herein, the G and the L ones have a frequency of 15.6%. Achilli et al. (2012) reported a frequency of 8.3% for haplogroup G and 22.4% for haplogroup L in the Middle East. This haplogroup (L) is considered ancient, as it has been reported in horses in the Iberian Peninsula since the Neolithic Bronze Age (McGahern et al., 2006; Cieslak et al., 2010; Achilli et al., 2012).

## Conclusion

This study evaluated the genetic variability of the AKH horse bred in Italy since 2000. Despite the low number of horses and the use of few stallions, during these 17 years, a large genetic variability with a low degree of inbreeding has maintained the breed over the years, confirming a well-balanced breeding practice. In fact, microsatellite markers showed a high level of heterozygosity that was comparable to findings in AKH populations reared in other countries.

The mtDNA analyses on the Italian AKH confirmed the breed’s high matrilineal variability, and added four new haplotypes that were never described before in the breed. The results also confirmed the close genetic relationship with the other AKH populations and the Middle Eastern horses represented by Arabian horse, Caspian Pony and Barb horse breeds, estimated as one of the most ancient and primitive clades of *Equus caballus*.

##  Supplemental Information

10.7717/peerj.4889/supp-1 Table S1 GenBank accession numbers and the references for the 126 horse mtDNA sequences used in the present study

10.7717/peerj.4889/supp-2 Table S2 Linkage disequilibrium *P*-val (*P* < 0.005) estimated on Italian Akhal-Teke Chromosome position in cM of the 17 horse microsatellites (Sheet 1). Linkage disequilibrium *P*-val (*P* < 0.005) estimated on Italian Akhal-Teke population (all samples) (Sheet 2). Linkage disequilibrium *P*-val (*P* < 0.005) estimated on Founders Italian Akhal-Teke population (imported 1991–2000) and on New population of Italian Akhal-Teke (born after 2000) (Sheet 3).

10.7717/peerj.4889/supp-3 Table S3 Comparison between the AKH imported from 1991 to 2000 (Founders) and the current AKH population bred in Italy (New Population) using 16 microsatellites NSTS, number of microsatellites; NS, number of samples; NA, absolute number of alleles; MNA, Mean number of alleles; NE, effective number of alleles; *P*-val, genetic equilibrium according to HW; Ho, observed heterozygosity; He, expected heterozygosity; FIS, inbreeding coefficient.
*Akhal-Teke imported in the years from 1991 to 2000 (Founders).

10.7717/peerj.4889/supp-4 Table S4 Haplotype sequence variations and haplogroups definition on Italian Akhal Teke and Arabian Horse breeds and on 93 Genbank sequences All the sequences were aligned with the complete horse mtDNA sequences X79547 and JN398377, using the ClustalW algorithm implemented in BioEdit. Haplogroup definition was performed according to Achilli et al. (2012).
AKH, Akhal-Teke; ARH, Arabian Horse; BRH, Barb Horse; CAP, Caspian Pony; PRZ, Przewalskii horse.
* (Xu et al., 1997): reference used for alignemet of sequences.
^b^ Achilli et al., 2012: reference used for haplogroup definition.

10.7717/peerj.4889/supp-5 Table S5 Matrix of pairwise FST The matrix of pairwise population genetic differentiation (*F*_*ST*_) was calculated based on mtDNA sequences and was used to evaluate genetic differentiation among Italian AKH, Italian Arabian, and the 93 horse GenBank sequences used.
AKH_ITY = Akhal-Teke and ARH_ITY = Arabian horse present study; CAP_a, Caspian Pony and AKH_d, Akhal-Teke horse Flanneryetal2003; CAP_b, Caspian Pony and BRH, Barb horse (Jansen et al., 2002); ARH_a, Arabian horse, AKH _a, Akhal-Teke horse and PRZ, Przewalskii horse (Achilli et al., 2012); AKH_b, Akhal-Teke horse (McGahern et al., 2006); AKH_c, Akhal-Teke horse (Lippold et al., 2011); AKH_e, Akhal-Teke horse (Priskin et al., 2010); Ref_seq, X79547 Xuetal1997 and JN398377 (Achilli et al., 2012).

10.7717/peerj.4889/supp-6 Table S6 Genotypes of the 95 Akhal-Teke horse bred in Italy The raw data are the genotype of the 95 samples of AKH bred in Italy. The 17 microsatellites are AHT4, AHT5, ASB2, ASB17, ASB23, CA425, HMS1, HMS2, HMS3, HMS6, HMS7, HTG4, HTG6, HTG7, HTG10, VHL20 and LEX03. Data are in GENEPOP format (3-Digit Alleles format). Data are in GENEPOP format (3-Digit Alleles format). The microsatellite LEX03 maps on X chromosome. The females are homozygous or heterozygous at the locus, whereas the males are hemizygous.

## Additional Information and Declarations

### DNA Deposition

The following information was supplied regarding the deposition of DNA sequences: GenBank: MF580226–MF580244.
