# Supplementary material for: Genetic variability of Akhal-Teke horses bred in Italy
Source: PeerJ. 2018 Sep 6;6:e4889. doi: 10.7717/peerj.4889 (PMC6129384; doi:10.7717/peerj.4889)
Supplement: Table S1 [file peerj-06-4889-s001.docx]

**Supplemental Table S1.**

**Table S1** GenBank accession numbers of the 126 horse mtDNA sequences used in the present study.

| **Accession number** | **N** | **Breed** | **Reference** |
| --- | --- | --- | --- |
| X79547, JN398377 | 2 | Ref. mtDNA samples | Xu et al., 1994; Achilli et al., 2012 |
| JN398385, JN398393, JN398404, JN398410, JN398422, JN398424, JN398435, JN398449-50, JN398452-53 | 11 | Akhal-Teke horse | Achilli et al. 2012 |
| JN398380, JN398392, JN398412, JN398434, JN398448 | 5 | Arab Horse | Achilli et al. 2012 |
| JN398402 - JN398403 | 2 | Przewalski's horse | Achilli et al. 2012 |
| DQ327950-67 | 18 | Akhal-Teke horse | McGahern et al 2006 |
| EU093045-63 | 19 | Akhal-Teke horse | Lippold et al. 2011 |
| AY246174-79 | 6 | Akhal-Teke horse | Flannery et al. 2003 unpub |
| AY246195-00 | 6 | Caspian Pony | Flannery et al. 2003 unpub |
| AJ413724-30 | 7 | Caspian Pony | Jansen et al. 2002 |
| AJ413649; AJ413658-71 | 15 | Barb horse | Jansen et al. 2002 |
| HQ439441-42 | 2 | Akhal-Teke horse | Priskin et al. 2010 |
| *MF580226-36* | *11* | *Akhal-Teke horse* | *Present study* |
| *MF580237-44* | *8* | *Arab horse* | *Present study* |

**References**

1. Achilli A, Olivieri A, Soares P, et al (2012) Mitochondrial genomes from modern horses reveal the major haplogroups that underwent domestication. Proc Natl Acad Sci USA. 109:2449–2454.
2. Flannery AR, Cothran EG (2003) Mitochondrial DNA sequence variation and the domestication pattern of horse Unpublished et al., 2003 unpub
3. Jansen T, Forster P, Levine MA, Oelke H, Hurles M, Renfrew C, Weber J, Olek K (2002) Mitochondrial DNA and the origins of the domestic horse. Proc Natl Acad Sci U S A. 99(16):10905-10
4. Lippold S, Matzke NJ, Reissmann M, Hofreiter M (2011) Whole mitochondrial genome sequencing of domestic horses reveals incorporation of extensive wild horse diversity during domestication. BMC Evol Biol 11:328
5. McGahern A, Bower MA, Edwards CJ, et al (2006) Evidence for biogeographic patterning of mitochondrial DNA sequences in Eastern horse populations. Anim Genet 37:494-497
6. Priskin K, Szabó K, Tömöry G, Bogácsi-Szabó E, Csányi B, Eördögh R, Downes CS, Raskó (2010) Mitochondrial sequence variation in ancient horses from the Carpathian Basin and possible modern relatives. Genetica 138(2):211-8
7. Xu X, Arnason U (1994) The complete mitochondrial DNA sequence of the horse Equus caballus: extensive heteroplasmy of the control region. Gene 148:657–662.
