# Supplementary material for: Genetic variability of Akhal-Teke horses bred in Italy
Source: PeerJ. 2018 Sep 6;6:e4889. doi: 10.7717/peerj.4889 (PMC6129384; doi:10.7717/peerj.4889)
Supplement: Table S3 — NSTS, number of microsatellites; NS, number of samples; NA, absolute number of alleles; MNA, Mean number of alleles; NE, effective number of alleles; P-val, genetic equilibrium according to HW; Ho, observed heterozygosity; He, expected heterozygosity; FIS, inbreeding coefficient. *Akhal-Teke imported in the years from 1991 to 2000 (Founders). [file peerj-06-4889-s003.docx]

| **Breed** | **NSTS** | **NS** | **NA** | **MNA** | **NE** | **H_o_** | **H_e_** | **F_IS_** |
| --- | --- | --- | --- | --- | --- | --- | --- | --- |
| Akhal-Teke Italy* | 16 | 20 | 94 | 5.9 | 3.40 | 0.672 | 0.684 | -0.009 |
| Akhal-Teke Italy | 16 | 75 | 100 | 6.2 | 3.04 | 0.633 | 0.642 | 0.008 |
